# Editorial: Clinical and genetic determinants of diabetes and complications

**DOI:** 10.3389/fendo.2023.1245837

**Published:** 2023-07-04

**Authors:** Sen Li, Maurizio Delvecchio, Kunka Mohanram Ramkumar, Xiaowen Mao, Xiaodong Sun, Shuzhen Guo

**Affiliations:** ^1^ School of Life Sciences, Beijing University of Chinese Medicine, Beijing, China; ^2^ Metabolic Disorders and Diabetes Unit, “Giovanni XXIII” Children’s Hospital, Azienda Ospedaliero-Universitaria (AOU) Policlinico-Giovanni XXIII, Bari, Italy; ^3^ Department of Biotechnology, School of Bioengineering, SRM Institute of Science and Technology, Kattankulathur, Tamil Nadu, India; ^4^ State Key Laboratory of Quality Research in Chinese Medicine, Institute of Chinese Medical Sciences, University of Macau, Macau, Macau SAR, China; ^5^ Department of Endocrinology and Metabolism, Affiliated Hospital of Weifang Medical University, Weifang, China; ^6^ School of Traditional Chinese Medicine, Beijing University of Chinese Medicine, Beijing, China

**Keywords:** diabetes, diabetes complications, risk factors, genetic basis, biomarkers

Diabetes Mellitus (DM) continues to be a significant cause of death worldwide, imposing a substantial burden on global public health. According to the data from International Diabetes Federation, the number of DM patients is expected to increase by 50% by 2030 compared to the 366 million cases reported in 2011. DM gives rise to various complications, resulting in organ damage, such as the heart and kidneys, ultimately leading to a diminished quality of life and an increased rate of premature mortality. For instance, individuals with diabetes have a twofold higher risk of cardiovascular mortality. The development of DM involves multiple factors, and several clinical risk factors, including overweight or obesity, have been suggested. However, the impact of several other potential factors on DM’s pathogenesis remains inconclusive. At the genetic level, having a family history of DM elevates the risk of developing the condition, and more than 500 genetic loci have been identified as being associated with DM. Early efforts to find genes associated with diabetes complications relied on family linkage analyses, candidate gene studies susceptible to false positives, and underpowered genome-wide association studies (GWAS) constrained by sample size. Detecting individuals who are very vulnerable to the disease may help with disease prevention. Nevertheless, the genetic determinants of DM complications are not yet well comprehended.

This Research Topic encompasses a collection of 30 studies that explore various aspects of diabetes and its complications. Specifically, it includes 15 studies examining the epidemiological characteristics and risk factors associated with diabetes and its complications. Furthermore, five studies analyze potential biochemical markers relevant to the pathogenesis of diabetes and diabetic complications, and seven studies evaluate genetic information for predicting diabetes and its complications, and three studies that assess treatment options.

The incidence and death rates associated with diabetic complications differ based on the population and the underlying factors contributing to the disease. For instance, a cross-sectional study conducted by Bundó et al. revealed a lower prevalence of diabetic foot disease in Catalonia (Spain) compared to previous similar studies. Meanwhile, in a systematic review and meta-analysis by Akhtar et al., the prevalence of foot ulcers in diabetic patients in Pakistan was investigated, indicating a relatively high prevalence of diabetic foot ulcers in the country. Alizadeh et al. conducted a cohort study involving 1329 participants aged 20 to 70 years with prediabetes, finding that the risk of progressing to diabetes was elevated in individuals with combined impaired fasting glycemia (IFG)/impaired glucose tolerance (IGT) compared to IFG alone. The results of a study by Liu et al. suggested that the OTUD3 gene variant rs78466831 is associated with type 2 DM (T2DM) and may serve as a risk factor for diabetic retinopathy. In another Chinese follow-up study, Shi et al. revealed that frailty is common among older adults with diabetes and is correlated with an elevated risk of adverse health outcomes.

Abnormalities in glucose and lipid metabolism play a crucial role in the progression of diabetic complications. Xiao et al. discovered that bile acids independently contribute to adverse renal outcomes in patients with diabetic kidney disease (DKD). Song et al. observed higher levels of remnant cholesterol in T2DM patients with the peripheral arterial disease (PAD), which were independently associated with the severity of PAD. In their study, Guan et al. compared the circulating adiponectin levels in Japanese women with varying levels of physical activity. They found that adiponectin primarily correlated with regional adiposity and high-density lipoprotein cholesterol (HDL-C). Li et al. summarized in their review that obesity can induce oxidative stress, which can contribute to insulin resistance, inflammation, and disorders in lipid metabolism, ultimately impacting cognitive dysfunction in individuals with diabetes. According to the findings of Lin et al., admission hyperglycemia in critically ill sepsis patients with diabetes was not found to be a contributing factor to the short-term prognosis.

With the increasing prevalence of diabetes, there is a proportional rise in the incidence of diabetic complications. Within this Research Topic, numerous papers explore the causal association between diabetes and its associated complications. Hao et al. provided evidence of a causal association between T2DM and systolic blood pressure. Guo et al. demonstrated a causal association between T2DM and coronary artery disease in East Asians but not atrial fibrillation. Previous research has identified a bidirectional link between nonalcoholic fatty liver disease (NAFLD) and T2DM. Yu et al. revealed the causal effect of NAFLD on the development of T2DM, emphasizing the need for further verification regarding the lack of a causal association between T2DM and NAFLD. Xu et al. indicated that lymphoid leukemia increases the risk of developing diabetes. Guo et al. suggested that T2DM is an independent risk factor for elevated risk of synovitis and tenosynovitis.

Biomarkers play a crucial role in the identification, diagnosis, prevention, and treatment monitoring of diseases. Since many complications of diabetes are difficult to detect, the discovery of biomarkers is essential for early detection and management. In a retrospective observational study, Song et al. identified that the combination model of the neutrophil/HDL-C ratio and the systemic inflammation response index was the most valuable in predicting PAD in individuals with T2DM. In another retrospective study, Li et al. suggested that the triglycerides/HDL-C ratio could be an effective marker for assessing the risk of NAFLD in patients newly diagnosed with T2DM. Mitra et al.‘s review summarized the prospective potential of exosomal microRNAs in diagnosis and clinically prognosis of gestational DM (GDM) and its impact on pregnancy outcomes. Huo et al. conducted a cross-sectional study, revealing that increased levels of circulating glycoprotein non-metastatic melanoma protein B are associated with both DM and cataracts, thus serving as a potential biomarker for DM-associated cataracts. Ferraz et al. suggested that 41 miRNAs were differentially regulated between T1DM and control individuals. In particular, hsa-miR-26b-5p and hsa-miR-21-5p may influence nuclear and mitochondrial dysfunction, leading to dysregulation in type 1 DM.

The progression of T2DM varies significantly and can be influenced by genetic factors. Therefore, numerous studies have explored genetic information related to diabetic complications in this field. Wang et al. conducted a cross-sectional study involving 120 T2DM patients from Han and Tibetan ethnic groups, revealing subtle differences in clinical characteristics between various ethnic groups that may be associated with epigenetic modifications. Liu et al. reviewed the association between epigenetic changes and DKD, emphasizing that DNA methylation, histone modification, and changes in noncoding RNA expression profiles are deeply involved in DKD-related inflammation, oxidative stress, hemodynamics, and abnormal signaling pathways. Ramos-Levi et al. identified a core set of single nucleotide polymorphisms (SNPs) associated with diabetes and GDM, suggesting the usefulness of identifying these genetic variants for designing preventive strategies, even in nutritional interventions. In their research, Mansour et al. performed an Exome-Wide Association Study on Emirati individuals diagnosed with T2DM. Through their study, they identified specific genetic loci that are linked to various categories of T2DM-related complications within the Emirati population. Zhang et al. investigated the distribution pattern of the CYP2C9 gene in Chinese Han individuals and identified variants that may impact drug metabolic activities. Yu et al. reported one colocalized locus and 14 additional candidate loci shared between T2DM and periodontal disease (PD)/oral health. Zhang et al. revealed that the MUC5B SNP rs2943512 (A > C) or the up-regulation of MUC5B in bronchial epithelial cells might significantly promote interstitial lung disease in patients with T2DM.

In this Research Topic, there are also papers focused on treating diabetic complications, aiming to improve the management and control of their progression, considering their high rates of disability and fatality. In a randomized controlled trial conducted in China, Cai et al. demonstrated that a subcutaneous administration of polyethylene glycol loxenatide, along with regular treatment, led to a more significant weight reduction than metformin in overweight or obese patients with T2DM. Akiyama et al.‘s review highlighted that SGLT2 inhibitors reduce blood glucose levels and decrease the likelihood of being admitted to the hospital due to heart failure and worsening renal function in patients with T2DM. Lastly, in a mini-review, Renuka et al. discussed the use of stimuli-responsive nanocomposite scaffolds in addressing specific issues related to wound healing and angiogenesis in diabetic patients, demonstrating their potential to interact with wound microenvironment, release bioactive materials in a regulated manner, and act as dressings for diabetic wound healing.

The Research Topic underscores the significance of clinical and genetic factors in the progression of diabetes and its complications, which holds important implications for prevention and treatment strategies. These findings provide valuable insights for clinical practice.

## Author contributions

All authors listed have made a substantial, direct, and intellectual contribution to the work and approved it for publication.

